# Editorial: Metabolic Flexibility

**DOI:** 10.3389/fnut.2022.946300

**Published:** 2022-06-02

**Authors:** Jose E. Galgani, Audrey Bergouignan, Jennifer Rieusset, Cedric Moro, Julie-Anne Nazare

**Affiliations:** ^1^Nutrition & Dietetics-Department of Health Sciences, Pontificia Universidad Católica de Chile, Santiago, Chile; ^2^Department of Nutrition, Diabetes and Metabolism, Faculty of Medicine, Pontificia Universidad Católica de Chile, Santiago, Chile; ^3^National Center for Scientific Research (CNRS), Pluridisciplinary Institute Hubert Curien (IPHC), University of Strasbourg, Strasbourg, France; ^4^Division of Endocrinology, Metabolism and Diabetes, Anschutz Health & Wellness Center, University of Colorado, Aurora, CO, United States; ^5^Univ-Lyon, CarMeN Laboratory, Inserm, Inrae, Université Claude Bernard Lyon-1, Lyon, France; ^6^Institute of Metabolic and Cardiovascular Diseases, Team MetaDiab, Inserm/Paul Sabatier University, Toulouse, France; ^7^Centre de Recherche En Nutrition Humaine Rhône-Alpes, Univ-Lyon, CarMeN Laboratory, Hospices Civils de Lyon, Cens, Université Claude Bernard Lyon1, Lyon, France

**Keywords:** fuel oxidation, respiratory quotient, insulin resistance, insulin sensitivity, metabolic syndrome, type-2 diabetes

Metabolic flexibility is defined as the capacity to switch among energy substrates to generate ATP depending on the physiological circumstances. Because ATP turnover is high and ATP reserve small, such capacity to generate ATP from different sources allows eukaryotic cells to survive in conditions of fluctuating fuel supply. At the whole-body level, the transition from fasting to feeding states determines cyclic changes in circulating and tissue fuel availability. Metabolic flexibility becomes crucial in adapting fuel oxidation to such transient oscillations in fuel supply. On the one hand, increased glucose supply (e.g. after ingesting carbohydrates) boosts glucose oxidation while suppressing fatty acid oxidation. On the other hand, the absence of exogenous glucose (e.g. overnight fast) increases fat mobilization. As a result, a higher fatty acid supply to tissues replaces glucose as an energy source. The coordinated switch among energy fuels according to fuel availability is the core concept defining metabolic flexibility.

Classically, metabolic flexibility has been determined by the extent to which the respiratory quotient (an index of the proportion of carbohydrate and fat being oxidized for energy) increases from fasting to glucose/insulin-stimulated conditions (e.g. clamp, meal test challenge). Pioneer work by Kelley et al. (1) measured metabolic flexibility by using the euglycemic-hyperinsulinemic clamps. They noted that individuals with obesity or type-2 diabetes manifested impaired metabolic flexibility compared with their lean, healthy counterparts. Such results prompted the notion that impaired metabolic flexibility could lead to insulin resistance and metabolic alterations. Contemporary studies also highlighted a strong inverse association between insulin sensitivity and ectopic lipid content (2). This latter finding reinforced the idea that a mismatch between fuel oxidation and fuel availability (i.e. impaired metabolic flexibility) underlies insulin resistance.

This Research Topic in Metabolic Flexibility aimed to attract studies providing insights about determinant factors of metabolic flexibility and the health consequences of being metabolically inflexible. In this regard, Glaves et al. conducted a systematic review on the association between adipose tissue features (e.g. total amount, distribution) and metabolic flexibility. Such research work faced the challenge of comparing studies that conceived different settings and markers of metabolic flexibility. This heterogeneity in assessing metabolic flexibility has dampened the possibility of conducting comprehensive inter-study data analysis, for instance, through meta-analyses, a crucial tool to ponder the overall relevance of individual studies. From this systematic review, Glaves et al. noted that adipose tissue total amount, waist circumference, and visceral fat were associated with metabolic flexibility, mainly when assessed by using the classic euglycemic clamp method. Which functional aspects of adipose tissue can directly influence and explain such associations remain elusive. One may speculate that adipose tissue amount, distribution, or both could influence circulating adipokines and metabolites, affecting oxidative tissue capacity and tissue fuel availability (i.e. metabolic flexibility). Kalafati et al. conducted additional work on adipose tissue by taking advantage of publicly available datasets. They assessed the contribution of relative macrophage frequencies on overall subcutaneous adipose tissue gene expression. Their analysis showed that subcutaneous adipose tissue from donors with high macrophage frequencies displayed increased inflammatory gene expression profile and decreased gene expression of lipid- and mitochondrial respiration-related pathways. From these studies, one may speculate that impaired metabolic flexibility could be one of the systemic consequences of such differential enrichment in adipose tissue macrophages.

Another study of this Research Topic (Fernández-Verdejo et al.) assessed volunteers of similar sex and age, who had the same excess body weight and composition, but contrasted metabolic flexibility to an oral glucose load. In general, such contrasting metabolic flexibility did not associate with differences in metabolic health. However, metabolically flexible vs. inflexible individuals showed elevated abdominal subcutaneous fat depot and faster blood triglyceride clearance. This study may support the notion that adipose tissue fat storage capacity determines metabolic flexibility and health, as demonstrated in mice (3).

Lactate captured attention in two publications included in this Research Topic, where lactate may serve as a potential circulating biomarker of metabolic flexibility. Still, more elaborated studies are required to understand lactate dynamics and its link to metabolic flexibility. The review by Zeng et al. reports on pyruvate dehydrogenase as a metabolic hub controlling lactate production. They proposed that pyruvate dehydrogenase could be a potential therapeutic target that can render benefit in extreme metabolic conditions such as sepsis. Lessons from that model may eventually translate to less extreme metabolic conditions. Lactate was also the focus of study in the original publication by San-Millan et al. The authors speculated that high lactate concentration might impair metabolic flexibility. They based that idea on the observation that chronic lactate exposure decreased mitochondrial fatty acid oxidation in a rat cardiomyocyte model.

These studies of diverse nature and focus somehow reflect our current knowledge regarding the role of metabolic flexibility on health outcomes. There is a need to define a setting and a validated set of metabolic flexibility biomarkers to enable comparability among studies. Such biomarkers and appropriate analysis should dissect inter-individual differences in metabolic flexibility from known confounders (e.g. fuel availability, baseline fuel oxidation, energy balance) (4). Such knowledge should translate into reliable and feasible biomarkers applicable to cohort and interventional studies to assess the relationship between metabolic flexibility and disease risk. We hope this Research Topic will encourage the scientific community to bring insightful studies in those areas.

## Author Contributions

JG wrote the first draft and finalized the submitted version. All authors revised and edited the manuscript and approved the submitted version.

## Conflict of Interest

The authors declare that the research was conducted in the absence of any commercial or financial relationships that could be construed as a potential conflict of interest.

## Publisher's Note

All claims expressed in this article are solely those of the authors and do not necessarily represent those of their affiliated organizations, or those of the publisher, the editors and the reviewers. Any product that may be evaluated in this article, or claim that may be made by its manufacturer, is not guaranteed or endorsed by the publisher.
